# Anti-tetherin activities in Vpu-expressing primate lentiviruses

**DOI:** 10.1186/1742-4690-7-13

**Published:** 2010-02-18

**Authors:** Su Jung Yang, Lisa A Lopez, Heiko Hauser, Colin M Exline, Kevin G Haworth, Paula M Cannon

**Affiliations:** 1 Department of Molecular Microbiology and Immunology, Keck School of Medicine of the University of Southern California, Los Angeles, California, USA

## Abstract

**Background:**

The anti-viral activity of the cellular restriction factor, BST-2/tetherin, was first observed as an ability to block the release of Vpu-minus HIV-1 from the surface of infected cells. However, tetherin restriction is also counteracted by primate lentiviruses that do not express a Vpu protein, where anti-tetherin functions are provided by either the Env protein (HIV-2, SIVtan) or the Nef protein (SIVsm/mac and SIVagm). Within the primate lentiviruses, Vpu is also present in the genomes of SIVcpz and certain SIVsyk viruses. We asked whether, in these viruses, anti-tetherin activity was always a property of Vpu, or if it had selectively evolved in HIV-1 to perform this function.

**Results:**

We found that despite the close relatedness of HIV-1 and SIVcpz, the chimpanzee viruses use Nef instead of Vpu to counteract tetherin. Furthermore, SIVcpz Nef proteins had activity against chimpanzee but not human tetherin. This specificity mapped to a short sequence that is present in the cytoplasmic tail of primate but not human tetherins, and this also accounts for the specificity of SIVsm/mac Nef for primate but not human tetherins. In contrast, Vpu proteins from four diverse members of the SIVsyk lineage all displayed an anti-tetherin activity that was active against macaque tetherin. Interestingly, Vpu from a SIVgsn isolate was also found to have activity against human tetherin.

**Conclusions:**

Primate lentiviruses show a high degree of flexibility in their use of anti-tetherin factors, indicating a strong selective pressure to counteract tetherin restriction. The identification of an activity against human tetherin in SIVgsn Vpu suggests that the presence of Vpu in the ancestral SIVmus/mon/gsn virus believed to have contributed the 3' half of the HIV-1 genome may have played a role in the evolution of viruses that could counteract human tetherin and infect humans.

## Background

The release of HIV-1 and other enveloped viruses from the surface of infected cells is reduced by the activity of the interferon-inducible cell surface protein BST-2/CD317/HM1.24/"tetherin" [1-6]. The importance of overcoming this restriction for virus replication is reflected in the growing list of viral proteins that have been shown to possess anti-tetherin activities, with the primate lentiviruses in particular having evolved diverse approaches that include the HIV-1 Vpu, HIV-2 Env and certain SIV Nef and Env proteins [2,3,7-12].

Analyses of the interactions between tetherins from different primate species and the anti-tetherin proteins used by viruses that infect those hosts have revealed a high degree of specificity. For example, although all tetherins analyzed to date can block HIV-1 particle release as efficiently as human tetherin, non-human tetherins are usually insensitive to antagonism by the HIV-1 Vpu protein [9,10,12-14]. The determinants of the Vpu-tetherin interaction have been mapped to the transmembrane (TM) domain of tetherin [9,13,15,16]. Within Vpu, the TM domain has long been known to be required for efficient virus release [17,18] and is now known to play an important role in the Vpu-tetherin interaction [2,3], while the cytoplasmic tail of Vpu contains a β-TrCP binding domain comprising residues serine 52 and 56 and a positively charged hinge region at the start of the cytoplasmic domain which both contribute to its anti-tetherin activity [14,19,20]. In addition, specificity has been observed in the interaction between tetherins and SIV Nef proteins that depends on a short stretch of amino acids that is present in the cytoplasmic tail of primate tetherins such as chimpanzee, macaque, or African green monkey, but not in human tetherin [9,10].

The primate lentiviruses have been classified into six major lineages on the basis of phylogenetic analyses (Table 1) [21,22]. Interestingly, only two lineages contain Vpu in their genome, the SIVcpz/HIV-1 lineage and certain members of the SIVsyk lineage that include the SIVgsn sublineage (SIVmus, SIVmon, and SIVgsn), as well as the SIVden isolate [23-28]. Vpu is a type I integral membrane protein that plays multiple roles in the HIV-1 life-cycle in addition to counteracting tetherin [29]. The close similarity between HIV-1 and SIVcpz led us to examine whether SIVcpz Vpu proteins could also counteract human tetherin, and if this could have been important in allowing HIV-1 to cross the species barrier and infect humans. Surprisingly none of the SIVcpz Vpu proteins that we tested had anti-tetherin activity, even against the species-matched chimpanzee tetherin. Instead, we found that an anti-tetherin activity in these viruses resides in the Nef protein. In contrast, the more distantly related SIVsyk viruses possessed an anti-tetherin activity in Vpu although, with a single exception, this was not active against human tetherin. Taken together, these findings suggest a high degree of flexibility in the evolution of anti-tetherin factors within the primate lentiviruses, with the diverse anti-tetherin strategies observed suggesting either convergent evolution or the re-acquisition of anti-tetherin activities in viral proteins as viruses adapted to new host species. It also leads us to speculate that having an anti-tetherin activity in Vpu in the SIVgsn ancestor that gave rise to the 3' half of the SIVcpz/HIV-1 genome may have been especially important for the evolution of the subgroup of viruses that could counteract human tetherin and infect humans.

**Table 1:** Anti-tetherin factors in primate lentiviruses (PLV)

PLV lineages	Vpu?	Anti-tetherin factors previously reported	Proteins analyzed in this study
SIVcpz/HIV-1[23,24,44]	+	HIV-1 Vpu[2,3]	HIV-1 NL4-3 Vpu
			SIVcpz GAB1 Vpu
			SIVcpz ANT Vpu
			SIVcpz MT145 Env/Vpu
			SIVcpz TAN3 Env/Vpu
			SIVcpz MB897 Env/Vpu, Nef
			SIVcpz EK505 Env/Vpu, Nef
			
SIVsm/mac/HIV-2[45,46]	-	SIVsm Nef[9,10]	
		SIVmac239 Nef[9,10]	SIVmac239 Nef
		HIV-2 ROD10 Env[7-9,50]	HIV-2 ROD10 Env
			
SIVagm[47]	-	SIVsab Nef[10]	
		SIVtan Nef[10,11]	
		SIVtan Env[11]	
			
SIVsyk[48]	+^1^	SIVmus Vpu^2 ^[11]	SIVmus 01CM2500 Vpu
			SIVmon 99CMCML1 Vpu
			SIVgsn 99CM71 Vpu
			SIVden Vpu
			
SIVl'hoest[49]	-	N/D	
SIVcol[21]	-	N/D	

^1 ^Vpu present only in SIVmus/mon/gsn sub-lineage and SIVden

^2 ^Activity reported for calculated ancestral sequence derived from four SIVmus Vpu sequences

## Results

### Vpu from SIVcpz does not counteract human or chimpanzee tetherin

**Figure 1:**
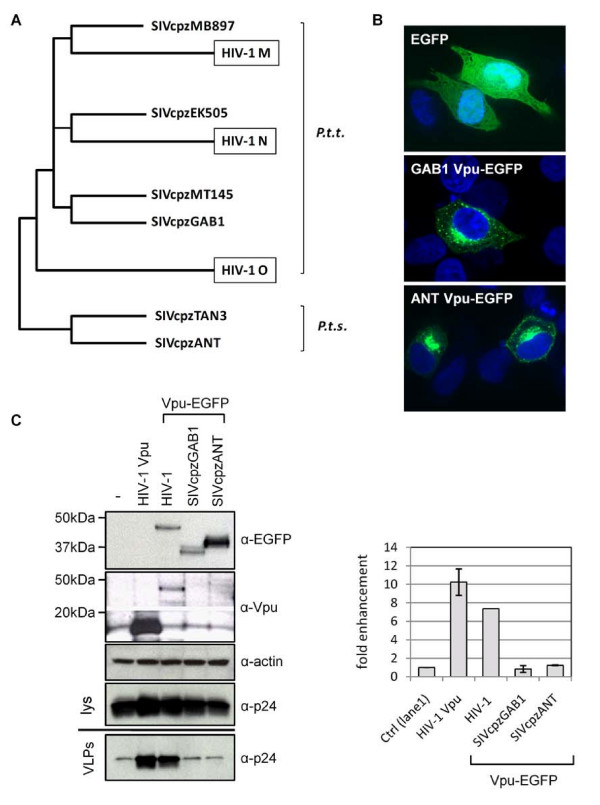
**HIV-1 but not SIVcpz Vpu overcomes human tetherin restriction**. (A) SIVcpz/HIV-1 lineage of the primate lentiviruses, showing three major HIV-1 groups (M, N and O) and the SIVcpz isolates used in this study. SIVcpz TAN3 and ANT were isolated from *Pan troglodytes schweinfurthii *(*P.t.s*.) and are less closely related to HIV-1 than SIVcpz strains isolated from *Pan troglodytes troglodytes *(*P.t.t*.). Figure adapted from Wain *et al*. (2007) [35]. (B) Confocal analysis of distribution of GAB1 and ANT Vpu-EGFP fusion proteins and EGFP control, in transiently transfected HeLa cells. (C) HeLa cells (express tetherin) were transfected with pHIV-1-pack (expresses HIV-1 Gag-Pol, Rev), together with either a control CMV expression vector (-), or expression plasmids for human codon-optimized Vpu from HIV-1 (HIV-1 Vpu), or non-codon-optimized EGFP tagged Vpu proteins from HIV-1, SIVcpz GAB1 or SIVcpz ANT. Cell lysates (lys) were probed with indicated antibodies. The Vpu-EGFP proteins from HIV-1, SIVcpz GAB1 and SIVcpz ANT have predicted molecular weights of 47, 33 and 42 kDa, respectively. Intracellular Gag proteins in cell lysates and virus-like particles released into supernatant (VLP) were detected using anti-p24 antibody. Mean-fold enhancement of HIV-1 VLP release in presence of Vpu is shown relative to baseline (control) levels in absence of Vpu for three independent experiments, except for the HIV-1 Vpu-EGFP sample (n = 1).

SIVcpz viruses are closely related to HIV-1 and contain a Vpu open-reading frame (Figure 1A, Table 1). We tested whether SIVcpz Vpu proteins have anti-tetherin activity by examining their ability to increase the release of HIV-1 virus like particles (VLPs) from HeLa cells, which naturally express human tetherin [2,3]. We initially tested the Vpu protein from the GAB1 strain of SIVcpz, which is representative of viruses isolated from *Pan troglodytes troglodytes *that are more closely related to HIV-1, and also the Vpu protein from SIVcpz ANT, which is representative of the more distantly related viruses isolated from *Pan troglodytes schweinfurthii *(Figure 1A) [24,30]. Both Vpu proteins were obtained as EGFP fusion proteins, whose functionality in CD4 down-regulation assays had previously been demonstrated [31]. Confocal microscopy revealed that the cellular distribution of GAB1 and ANT Vpu-EGFP (Figure 1B) resembled that which has been reported for HIV-1 Vpu [31,32]. In addition, both GFP and YFP fusion proteins of HIV-1 Vpu have previously been shown to retain anti-tetherin activity [2,19,33], and we confirmed the lack of effect of a C-terminal EGFP tag for HIV-1 Vpu by comparing the ability of Vpu and Vpu-EGFP to increase HIV-1 VLP release when expressed in HeLa cells (Figure 1C). Both proteins increased VLP release, and although the untagged Vpu construct had greater overall activity, this is likely a consequence of the higher levels of expression from this human codon-optimized construct. In contrast, neither of the two SIVcpz Vpu proteins had any effect on VLP release.

**Figure 2:**
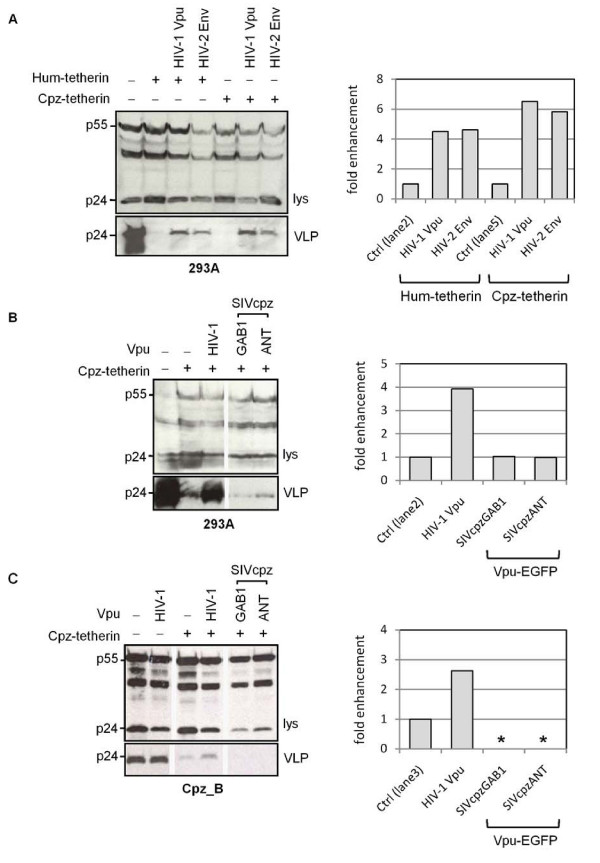
**Activity of HIV-1 and SIVcpz Vpu against chimpanzee tetherin**. Anti-tetherin activities of indicated viral proteins were examined by measurement of HIV-1 VLP release, detected by Western blotting of cell lysate and VLP fractions with anti-p24 antibody (left panels) or as mean-fold enhancement of VLP release relative to baseline (control) levels in absence of Vpu or Env proteins (right panels): (A) HIV-1 Vpu and HIV-2 Env activity against human (Hum) and chimpanzee (Cpz) tetherin expressed in 293A cells, (B) Activity of HIV-1 Vpu and SIVcpz GAB1 and SIVcpz ANT Vpu-EGFP proteins against Cpz-tetherin expressed in 293A cells, and (C) Activity of HIV-1 Vpu and SIVcpz GAB1 and SIVcpz ANT Vpu-EGFP proteins against Cpz-tetherin expressed in chimpanzee (Cpz_B) cells. * indicates p24 signal was too low to quantify.

Since species specificities have been noted in the interaction between tetherin and viral anti-tetherin factors [9-11,13-15], we next examined whether the SIVcpz Vpu proteins had activity against chimpanzee tetherin. We expressed chimpanzee tetherin in human 293A cells which, similar to other derivatives of 293 cells, do not constitutively express human tetherin [2,3]. We found that chimpanzee tetherin was able to suppress HIV-1 VLP release just as efficiently as human tetherin. Furthermore, chimpanzee tetherin restriction was antagonized to a similar extent as human tetherin by both the HIV-1 Vpu and HIV-2 Env proteins (Figure 2A). However, neither of the SIVcpz Vpu proteins was able to increase HIV-1 VLP release in the presence of chimpanzee tetherin (Figure 2B). To rule out any requirements for other chimpanzee cellular factors, we also repeated these analyses by expressing chimpanzee tetherin in a chimpanzee B cell line. Although the HIV-1 Vpu protein remained active against chimpanzee tetherin in this cell line, neither of the SIVcpz Vpus had any activity (Figure 2C). Taken together, these results indicate that the Vpu protein from SIVcpz is not an antagonist of either human or chimpanzee tetherin.

### SIVcpz Env does not have anti-tetherin activity

**Figure 3:**
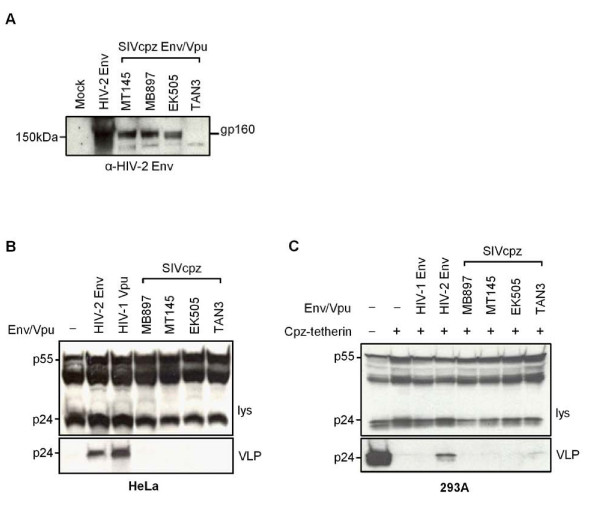
**Activity of SIVcpz genomic fragments against human and chimpanzee tetherin**. (A) Expression of Env proteins from SIVcpz subgenomic fragments (express Env, Vpu and Rev). (B) Activity of SIVcpz genomic fragments in HIV-1 VLP release assay against human tetherin present in HeLa cells. (C) Activity of SIVcpz genomic fragments in HIV-1 VLP release assay against chimpanzee (Cpz) tetherin expressed in 293A cells. HIV-1 Vpu, HIV-2 Env, and HIV-1 Env were included as positive and negative controls as indicated.

Other viral proteins in the primate lentiviruses that have been reported to have anti-tetherin activity include the Env proteins from HIV-2 and SIVtan [7,8,12] and the Nef proteins from certain SIVs [9-11]. We examined the possibility that SIVcpz Env had anti-tetherin activity by generating HIV-1 VLPs in the presence of fragments of SIVcpz genomes comprising the Env, Vpu, and Rev proteins, in a configuration that we have previously shown can lead to expression of all three HIV-1 and HIV-2 proteins [7]. We performed these analyses on four additional SIVcpz isolates, spread throughout the lineage, including MB897 and EK505 that are closely related to HIV-1 subtypes M and N respectively, as well as MT145 and TAN3 which are more distantly related (Figure 1A) [34,35]. Due to the lack of specific antisera against these Env and Vpu proteins, we were only able to confirm the expression of three out of four Env proteins from these constructs (Figure 3A). None of the genomic fragments we tested exhibited anti-tetherin activity, either against endogenous human tetherin present in HeLa cells (Figure 3B), or in 293A cells transfected with chimpanzee tetherin (Figure 3C). These data further confirm the lack of activity of the SIVcpz Vpu proteins and, additionally, reveal that SIVcpz Env proteins are not anti-tetherin factors.

### SIVcpz Nef counteracts chimpanzee but not human tetherin

**Figure 4:**
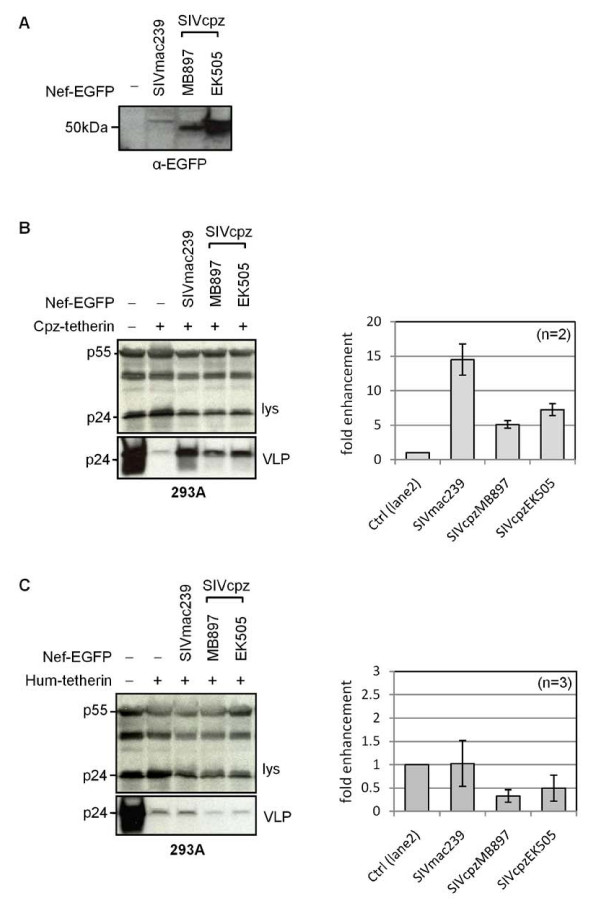
**Anti-tetherin activity of SIVcpz Nef**. (A) Expression of indicated SIV Nef-EGFP proteins, detected with anti-GFP antibody. Activities of Nef-EGFP proteins against (B) chimpanzee, or (C) human tetherin expressed in 293A cells. Mean fold-enhancement of HIV-1 VLP release in presence of Nef-EGFP is shown relative to baseline (control) levels for n = 2 or 3 independent experiments.

The absence of anti-tetherin activity in both SIVcpz Env and Vpu proteins led us to examine the Nef protein, since certain SIVs have previously been shown to have activity against tetherins from their host species that is a function of Nef (Table 1). We constructed Nef-EGFP proteins from SIVcpz MB897 and EK505, as well as from SIVmac239, since this has previously been reported to be active against macaque, but not human tetherin [9,10]. All three Nef-EGFP fusion proteins were expressed, although we noted that the SIVmac239 protein was present at lower steady-state levels (Figure 4A). We found that all three proteins were able to counteract chimpanzee tetherin when expressed in human 293A cells, with the least well expressed SIVmac239 protein having the greatest activity. This indicates that for the SIVcpz viruses, Nef fulfills the role of anti-tetherin factor (Figure 4B). In contrast, none of the Nef proteins had activity against human tetherin expressed in the same cells (Figure 4C). This finding agrees with observations recently reported by Sauter *et al*., who demonstrated that other SIVcpz isolates, including SIVcpzGAB1 and ANT, also contain a functional anti-tetherin activity in their Nef proteins [36].

**Figure 5:**
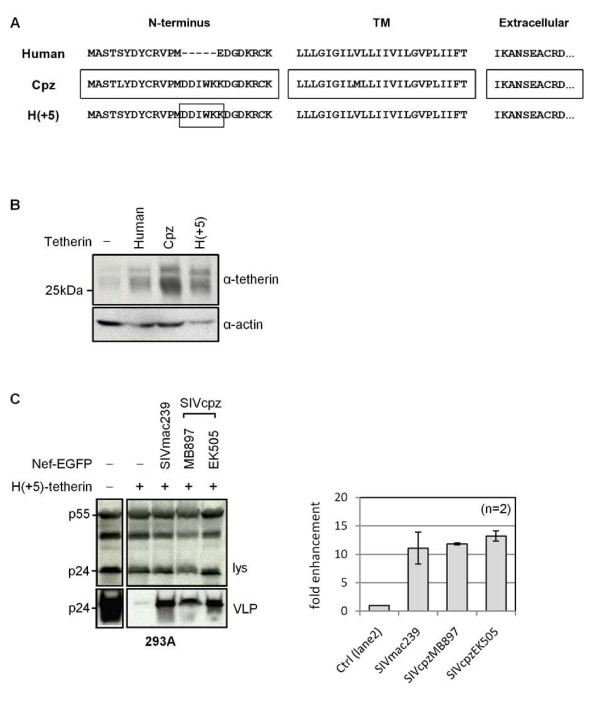
**Specificity of interaction between SIVcpz and SIVmac Nef proteins and tetherin**. (A) Partial sequence alignment of human, chimpanzee (Cpz) and H(+5)-tetherin proteins, showing the N-terminal cytoplasmic tail, the transmembrane (TM) domain and the start of the extracellular domain. H(+5)-tetherin contains an insertion (DDIWKK) from Cpz-tetherin in place of human tetherin residue E-14. (B) Western blot of expression of indicated tetherin constructs, from lysates of transfected 293A cells. (C) Anti-tetherin activities of SIVmac239 and SIVcpz Nef-EGFP proteins against H(+5)-tetherin expressed in 293A cells. Mean fold-enhancement of HIV-1 VLP release in presence of Nef-EGFP proteins is shown relative to baseline (control) levels for n = 2 independent experiments.

The specificity of SIVmac Nef for macaque, but not human tetherin, has previously been reported to be conferred by the presence of 5 additional residues in the cytoplasmic tail of macaque tetherin, G/D-DIWK [9,10]. We asked whether this same sequence was responsible for the specificity observed in the SIVcpz Nef proteins by creating a chimeric human tetherin containing an insert of the equivalent chimpanzee residues, H(+5)-tetherin (Figure 5A). We confirmed that H(+5) was expressed (Figure 5B) and fully functional in suppressing virus release when transfected into 293A cells (Figure 5C). The presence of these 5 additional residues was sufficient to make human tetherin a target for both SIVmac239 Nef and the SIVcpz Nef proteins (Figure 5C), indicating that the observed species specificity of the interaction between the SIVcpz Nef proteins and tetherin involves the same target sequence as SIVmac Nef.

### Anti-tetherin activity of SIVsyk Vpu

**Figure 6:**
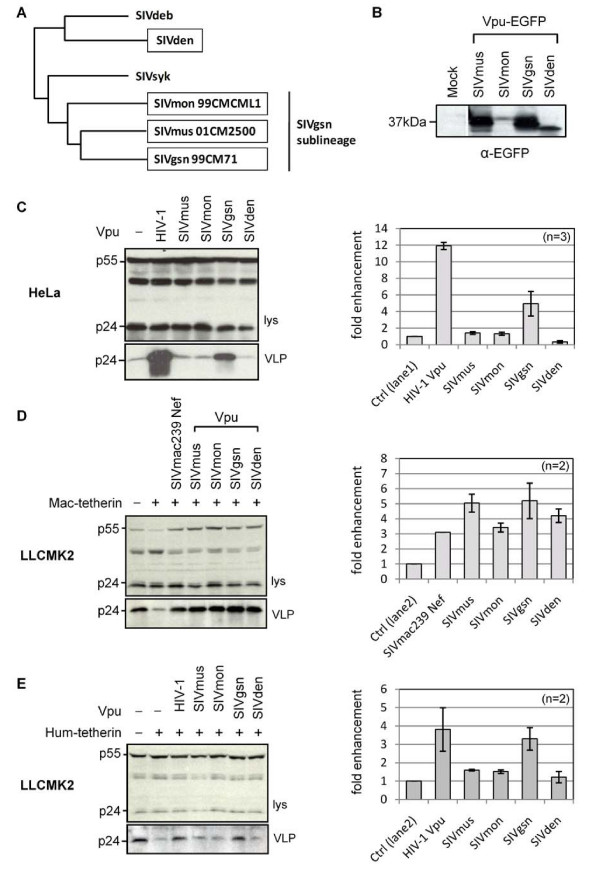
**Anti-tetherin activity of SIVsyk lineage Vpu proteins**. (A) SIVsyk lineage of the primate lentiviruses, showing viruses that express Vpu (boxed). SIVmon/mus/gsn viruses form the SIVgsn sublineage, while SIVden is less closely related. Figure adapted from Dazza *et al*. (2005) [28]. (B) Expression of SIVmus/mon/gsn and SIVden Vpu-EGFP proteins detected with anti-GFP antibody. Anti-tetherin activities of indicated Vpu-EGFP proteins were measured by HIV-1 VLP release assays, against (C) human tetherin present in HeLa cells, (D) Mac-tetherin expressed in macaque (LLCMK2) cells, and (E) Hum-tetherin expressed in LLCMK2 cells. HIV-1 Vpu and SIVmac239 Nef-EGFP proteins were included as controls. Mean fold-enhancement of HIV-1 VLP release in presence of anti-tetherin proteins is shown relative to baseline (control) levels, for n = 2 independent experiments.

Certain members of the SIVsyk lineage express Vpu, specifically those from the SIVmus/mon/gsn sub-lineage [25-27], and also the SIVden isolate [28] (Figure 6A). We analyzed the anti-tetherin activity of Vpu proteins from representatives of each of these four groups within the SIVsyk lineage. Each SIV Vpu was constructed as an EGFP fusion protein, and the expression of each protein was analyzed by Western blotting, where we observed some differences in steady-state levels (Figure 6B). We analyzed their activity against both human and macaque tetherin, since macaques are more closely related to the Old World primate hosts of these viruses. Macaque tetherin has previously been shown to be resistant to HIV-1 Vpu, but counteracted by SIVmac239 Nef [9,10] and HIV-2 Env [9], and we confirmed these specificities (data not shown). Anti-tetherin activity was assessed in both HeLa cells, (Figure 6C), and in macaque LLCMK2 cells transiently transfected with macaque tetherin (Figure 6D). We found that all four SIV Vpu proteins were able to counteract the inhibition of VLP release caused by macaque tetherin, which is in agreement with a recent report [36]. We cannot rule out that the greater activity of the SIVmus and SIVgsn Vpu-EGFP proteins could arise from their higher expression levels. In contrast, only the Vpu from SIVgsn (strain 99CM71) showed activity against human tetherin. Our finding of activity against human tetherin in this SIVgsn isolate is the first report of such an activity in a naturally occurring SIV Vpu protein.

To address whether the lack of activity of the Vpu proteins from SIVmus/mon/den in HeLa cells was caused by incompatibility between their Vpu proteins and human tetherin or, instead, reflected some other differences between human and macaque cells, we repeated these analyses expressing human tetherin in LLCMK2 cells. Similar to our findings in HeLa cells we observed that only SIVgsn Vpu had activity against human tetherin in this cell background (Figure 6E). SIVgsn 99CM71 Vpu is therefore an example of a naturally existing protein from a non-human primate lentivirus that has activity against human tetherin.

### Specificity of interaction between SIVsyk Vpu proteins and tetherin

**Figure 7:**
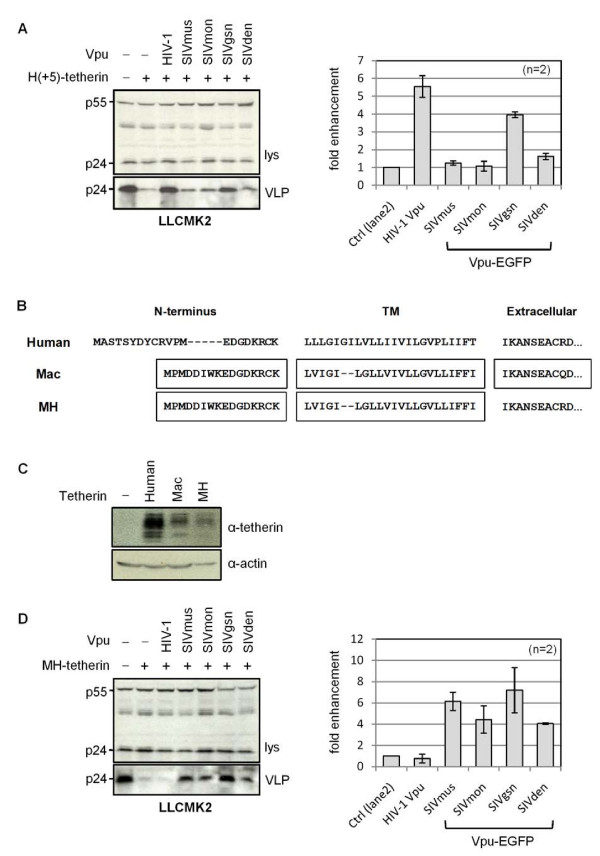
**Specificity of interaction of SIVsyk Vpu proteins and tetherin**. (A) Anti-tetherin activities of HIV-1 Vpu and indicated SIVsyk Vpu-EGFP proteins against H(+5)-tetherin expressed in LLCMK2 cells, measured by HIV-1 VLP release assay. Mean fold-enhancement of HIV-1 VLP release in the presence of anti-tetherin proteins is shown relative to baseline (control) levels in their absence, for n = 2 independent experiments. (B) Partial sequence alignment of human, macaque (Mac) and MH-tetherin showing the N-terminal cytoplasmic tail, the transmembrane (TM) domain and the start of the extracellular domain. Mac-tetherin starts at M-11 of the full-length *Macaca mulatta *tetherin [9]. MH-tetherin has N-terminal cytoplasmic tail and TM domains of Mac-tetherin with a human tetherin extracellular domain. (C) Western blot of expression of indicated tetherin constructs, from lysates of transfected 293A cells. (D) Anti-tetherin activities of HIV-1 Vpu and indicated SIVsyk Vpu-EGFP proteins against MH-tetherin expressed in LLCMK2 cells, measured by HIV-1 VLP release assay. Mean fold enhancement of VLP release in presence of Vpu proteins is shown relative to baseline (control) levels in absence of Vpu, for n = 2 independent experiments.

Since the SIVsyk Vpu proteins displayed species specificities in their interactions with tetherin, we asked whether this mapped to the same region of tetherin responsible for the specificity of interaction with SIVmac and SIVcpz Nef proteins, by examining their activity against the chimeric protein H(+5)-tetherin (Figure 5A). In contrast to the situation with the SIVcpz Nef proteins, where the addition of these amino acids to the cytoplasmic tail of human tetherin conferred sensitivity (Figure 5C), we found that the H(+5)-tetherin remained resistant to all of the SIVsyk Vpu proteins except SIVgsn (Figure 7A), which was unsurprising, given its activity against both human and macaque proteins (Figure 6).

The species-specificity of the interaction between the HIV1 Vpu protein and tetherin has been mapped to the TM domain of both proteins [2,3,9,13,15,16]. We therefore replaced both the N-terminal cytoplasmic tail and TM regions of human tetherin with the corresponding macaque sequences to create MH tetherin (Figure 7B) and confirmed protein expression (Figure 7C). We observed that all of the SIVsyk Vpu proteins, but not HIV-1 Vpu, had activity against this chimeric protein (Figure 7D). This suggests that, similar to the situation with HIV-1 Vpu, the specificity of the interaction between SIVsyk Vpu proteins and tetherin also maps to the TM domain.

### SIVgsn Vpu removes human tetherin from the surface of HeLa cells

**Figure 8:**
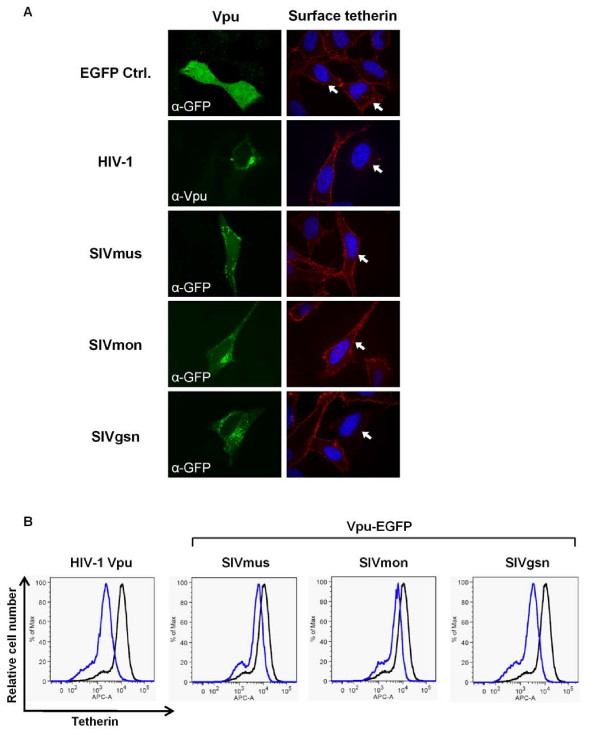
**Ability of Vpu proteins to remove human tetherin from surface of cells**. (A) HeLa cells were transfected with a control EGFP expression plasmid, HIV-1 Vpu or Vpu-EGFP fusion proteins from SIVmus/mon/gsn isolates. Non-permeabilized cells were incubated with anti-BST-2 antibody to reveal cell surface tetherin, followed by fixation and permeablization to visualize intracellular proteins. HIV-1 Vpu (untagged) was used as a positive control for tetherin removal from surface and visualized with an anti-Vpu antiserum, while the SIV Vpu-EGFP proteins and EGFP control were visualized using anti-GFP antibody. Both HIV-1 and SIVgsn Vpu proteins removed human tetherin from the cell surface, but SIVmus and SIVmon did not. Vpu-expressing cells are arrowed. (B) HeLa cells were transfected with a control EGFP expression plasmid alone or together with HIV-1 Vpu, or with each of the SIVmus/mon/gsn Vpu-EGFP expression plasmids. Cells were stained with anti-BST-2 antibody and analyzed by FACS. The histograms show cells gated for EGFP expression, with the control EGFP expression vector cells in black, and Vpu samples in blue.

We next asked whether the SIVgsn Vpu was able to remove tetherin from the cell surface, as we and others have previously observed for HIV-1 Vpu and human tetherin [3,14,16,20,33,37]. As controls we also included Vpu proteins from the SIVmus and SIVmon strains that were not active against human tetherin. Confocal analysis of cell surface tetherin on HeLa cells transfected with Vpu-EGFP fusion proteins demonstrated the removal of tetherin by only the HIV-1 and SIVgsn Vpu proteins (Figure 8A), and FACS analysis confirmed these observations (Figure 8B). Therefore, the ability to counteract human tetherin correlates with its removal from the cell surface for both HIV-1 and SIVgsn Vpu.

## Discussion

The primate lentiviruses exhibit a high degree of flexibility in their ability to counteract the BST-2/tetherin restriction of virus release. To date, three different proteins (Vpu, Env and Nef) have been shown to act as anti-tetherin factors in different primate lentiviruses, which highlights the importance of an anti-tetherin activity for their life-cycle. The HIV-1 Vpu protein is the prototypical anti-tetherin factor, and the observation that mature virions remain attached at the cell surface if tetherin is not counteracted was first observed for Vpu-minus HIV-1 [38]. The paradox of such an activity being associated with a protein that is not present in the genome of the other lineage of lentiviruses that infect humans, HIV-2, was resolved when a virus release-enhancing activity was mapped to the HIV-2 Env protein [8,39]. More recently, several different strains of SIV have been shown to carry anti-tetherin activities in either Nef or Env proteins [9-12,36].

The Vpu open-reading frame is not unique to HIV-1 but is also present in all other members of the SIVcpz/HIV-1 lineage. In addition, certain members of the SIVsyk viruses (the SIVgsn sublineage and SIVden) code for Vpu. We therefore asked the question, if Vpu is present, does it always exhibit anti-tetherin activity? Interestingly, despite the close relationship between HIV-1 and the SIVcpz viruses, we found that the Vpu proteins from SIVcpz do not have activity against either chimpanzee, macaque or human tetherin. Instead, we determined that these viruses have evolved to target chimpanzee tetherin using their Nef protein, a finding that has also recently been reported [36]. In this way, these viruses are more similar to other SIV strains, specifically SIVsm/mac and SIVagm, that also possess anti-tetherin activities in Nef [9-11]. Primate tetherins, including chimpanzee, macaque and African green monkey, differ from the human protein by having an additional sequence of 5 amino acids (G/D-DIWK) in their cytoplasmic tail, which has previously been shown to be necessary for SIVmac Nef to counteract primate tetherins [9,10]. Similarly, we have now confirmed that this motif is also essential for the recognition of primate tetherins by SIVcpz Nef proteins. It therefore seems likely that the use of Vpu by HIV-1 and Env by HIV-2 was necessitated, in part, because Nef cannot easily target human tetherin in the absence of this motif.

The fact that SIVcpz strains maintain the Vpu ORF, despite its lack of activity against chimpanzee tetherin, probably reflects the fact that Vpu is a multi-functional protein [29]. Indeed, down-regulation of CD4 by SIVcpz Vpu proteins has been confirmed by others [31,36,40]. Thus, Vpu's ability to target CD4 is well conserved in the SIVcpz/HIV-1 lineage, while the anti-tetherin activity may be a more recently acquired, or re-acquired, activity in the viruses that infect humans.

In contrast to the situation with SIVcpz, we found that Vpu proteins from the SIVsyk viruses are capable of antagonizing tetherin restriction. Within the SIVsyk lineage, there is a close phylogenetic relationship between the SIVmus/mon/gsn viruses, which form a separate sublineage termed SIVgsn [24,26,28]. A less closely related virus, SIVden, also expresses Vpu, although the protein is 10 amino acids shorter than Vpu from the SIVmus/mon/gsn viruses [28]. Representative Vpu proteins from all four groups of viruses were tested and found to be capable of overcoming the restriction mediated by macaque tetherin. Consistent with our observations, Lim *et al*. have also found that SIVmus Vpu antagonizes African green monkey and mustached monkey tetherins [11]. In addition, while this manuscript was in preparation, Sauter *et al*. also reported that SIVmus/mon/gsn Vpu proteins have anti-tetherin activities against host species-matched tetherins, with the specificity determined by the TM domain of tetherin [36].

An interesting finding from our studies of the SIVsyk lineage was that the Vpu protein from SIVgsn 99CM71 was also capable of antagonizing human tetherin. This activity was confirmed by both confocal studies and FACS analyses, where we observed that similar to the HIV-1 Vpu, SIVgsn Vpu removed tetherin from the surface of human cells. The presence of an anti-tetherin activity that is active against the human form of the protein in this SIVgsn virus supports the hypothesis that the SIVcpz/HIV-1 lineage arose by recombination, with the 5'-half of the genome originating from SIVrcm and the 3'-half, that includes Vpu, deriving from the SIVgsn sublineage [30,41]. Consequently, a recombinant ancestor of HIV-1 could have contained a Vpu protein with some capability of targeting human tetherin. At the same time, the lack of the G/D-DIWK motif in the cytoplasmic tail of human tetherin would have restricted the adoption of Nef for this activity, as occurred in the SIVcpz viruses. Since the interactions between Vpu and tetherin are highly specific, [9,10,13-15], it is likely that the acquisition of the ability to target human tetherin in HIV-1 Vpu would have resulted in the loss of any ability to target Old World primate tetherins, leading to the present day restriction of HIV-1 Vpu's activity for human tetherin.

## Conclusions

The ability to counteract tetherin restriction appears to be an essential activity of primate lentiviruses. At least three different proteins have evolved in different virus backbones and host environments to target this host cell restriction. Although several diverse SIVs use Nef to target tetherin, including the SIVcpz viruses that are closely related to HIV-1, the lack of a 5 residue sequence in the cytoplasmic tail of human tetherin makes it a difficult target for Nef. Possibly, this led to the adoption of alternate anti-tetherin approaches in the human immunodeficiency viruses based on Vpu (HIV-1) and Env (HIV-2). For HIV-1, we speculate that the presence of anti-tetherin activity in the Vpu protein from the SIVgsn ancestor that contributed the 3'-half of the SIVcpz/HIV-1 genome allowed HIV-1 to evolve such an activity in Vpu, and contributed to the ability of this virus to successfully infect humans.

## Methods

### Cell lines

HeLa and LLCMK2 (macaque) cells were obtained from the American Type Culture Collection; 293A cells were obtained from Qbiogene/MP Biomedicals (Irvine, CA). All cells were maintained in DMEM (Mediatech, Herndon, VA) supplemented with 10% fetal bovine serum (FBS) (Mediatech) and 2 mM glutamine (Gemini Bio-Products, West Sacramento, CA). The Cpz-B (chimpanzee) cell line was kindly provided by Jae Jung (University of Southern California) and cultured in RPMI media (Invitrogen, Carlsbad, CA) with 10% FBS and 2 mM glutamine.

### Plasmids

Plasmid pHIV-1-pack expresses HIV-1 Gag-Pol and Rev [7]. Plasmid pcDNA-Vphu (HIV-1 Vpu) encodes a human codon-optimized form of Vpu from HIV-1 isolate NL4-3, kindly provided by Klaus Strebel (NIH) [42]. Expression plasmids for the Env proteins from HIV-2 (isolate ROD10, HIV-2 Env) and HIV-1 isolate BH10 (HIV-1 Env) have been previously described [7]. Expression plasmids containing Vpu-EGFP fusion proteins from SIVcpz isolates GAB1 and ANT were kindly provided by Edward Stephens (University of Kansas) [31]. Vpu-EGFP fusion proteins from SIVmus 01CM2500 (GenBank: ABO61057), SIVmon 99CMCML1 (GenBank: AAR02384), SIVgsn 99CM71 (GenBank: AAM90227) and SIVden (GenBank: CAE46404) were synthesized as human codon-optimized open-reading frames (Bio Basic Inc. Ontario, Canada) and fused to EGFP by cloning into vector pAcEGFP-N1 (Clontech, Mountain View CA). Fragments of SIVcpz genomes spanning the Vpu-Env-Rev open reading frames (SIVcpz Env/Vpu) were PCR amplified and cloned into the immediate-early CMV promoter expression vector, pSA91. The SIVcpz isolates used were MT145 (GenBank: DQ373066; co-ordinates 5495 to 8436), MB897 (GenBank: EF535994; co-ordinates 5519 to 8369), EK505 (GenBank: DQ373065; co-ordinates 5522 to 8346), and TAN3 (GenBank: EF394358; co-ordinates 5603 to 8608), generously provided by Beatrice Hahn (University of Alabama). The Nef proteins from SIVmac239 (GenBank: AAU14056), SIVcpz MB897 (GenBank: ABU53024), and SIVcpz EK505 (GenBank: ABD19500) were cloned as Nef-EGFP fusion proteins in vector pAcEGFP-N1. A human BST-2/tetherin (Hum-tetherin) expression plasmid (pCMV6-XL5-Bst2) was obtained from Origene (Rockville, MD), and the corresponding tetherin from *Pan troglodytes *(Cpz-tetherin, GenBank: XP_512491), was generated by site-directed mutagenesis of the human protein. *Macaca mulatta *tetherin (Mac-tetherin, GenBank: ACV96781) starting at methionine-11, which corresponds to rBST2Δ10, and retains full tetherin activity and sensitivity to SIVmac239 Nef [9], was cloned by RT-PCR from total RNA isolated from LLCMK2 cells and inserted into vector pCMV6-XL5 (Origene). The chimeric tetherin H(+5)-tetherin is human tetherin with the sequence DDIWKK replacing amino acid E-14, while MH-tetherin comprises amino acids 11-38 of *M. mulatta *tetherin spliced to amino acid isoleucine-46 of human tetherin.

### Production and analysis of HIV-1 VLPs

HIV-1 VLPs were generated from HeLa, 293A, or LLCMK2 cells by transient transfection of 80-90% confluent cells in 10 cm plates with 8 μg of pHIV-1-pack, together with any additional specified plasmids, using Lipofectamine 2000 (Invitrogen, Carlsbad, CA). The following amounts of plasmid DNA were used: 2 μg of HIV-1 Vpu, HIV-1 BH10 Env, HIV-2 ROD10 Env, SIVcpz Env/Vpu, SIVmus/mon/gsn/den Vpu-EGFP; 5 μg of HIV-1, SIVcpzGAB1, or SIVcpzANT Vpu-EGFP; 0.5 μg of SIVmac239 and SIVcpz Nef-EGFP. Cpz_B chimpanzee cells were nucleofected using an Amaxa Nucleofector series X (Amaxa, South San Francisco, CA) using the pre-set program U-08. Cell lysates and viral particles were collected 24 or 48 hours post-transfection and analyzed by Western blotting, as previously described [43]. HIV-1 p24-reacting proteins were detected using rabbit HIV-1_SF2 _p24 Antiserum (AIDS Research and Reference Reagent Program, ARRRP) at a 1:3,000 dilution, followed by horseradish peroxidase (HRP)-conjugated goat anti-rabbit IgG (1:10,000) (Santa Cruz Biotechnology Inc., Santa Cruz, CA). Specific proteins were visualized using the enhanced chemiluminescence (ECL) detection system (Amersham International, Arlington Heights, IL). Exposed and developed films were scanned and quantified using the public domain NIH ImageJ software. The intensity of the CA-reacting bands on the Western blots was measured and the ratio of the signal in virions:lysates obtained. The fold-enhancement of virus budding was calculated by normalizing all values to the pHIV-1-pack only control.

### Western blotting

Expression of all Vpu-EGFP and Nef-EGFP fusion proteins was detected by Western blotting of cell lysates using rabbit anti-GFP at a 1:1000 dilution (Santa Cruz Biotechnology Inc.). Env expression from the SIVcpz subgenomic constructs was detected using rabbit anti-HIV-2 Env antiserum at a 1:1000 dilution (ARRRP). Expression of the various tetherin constructs was detected using rabbit anti-BST-2 at a 1:30,000 dilution (ARRRP, from Klaus Strebel). Actin was included as a loading control, and detected using a mouse anti-actin antibody (Sigma Aldrich, St. Louis, MO) at a dilution of 1:15,000. Secondary antibodies used were HRP-conjugated anti-rabbit IgG at a 1:10,000 (Santa Cruz Biotechnology Inc) or HRP-conjugated anti-mouse IgG at a 1: 10,000 (Sigma Aldrich).

### Confocal microscopy

HeLa cells were transfected with 2 μg of Vpu-EGFP expression plasmids in 10 cm plates, then 18-24 hours later, seeded on coverslips coated with poly-L-lysine (Sigma Aldrich). The cells were incubated for an additional 24 hours at 37°C, fixed with 4% paraformaldehyde for 20 minutes at room temperature, washed three times in PBS and subsequently permeabilized for 10 minutes in 0.1% Triton X-100 at room temperature, followed by washing three times in PBS. Processed cells were mounted in Prolong Gold antifade reagent with DAPI (Molecular Probes, Invitrogen). For analysis of tetherin surface expression, HeLa cells were transfected with 2 μg of HIV-1 Vpu or SIVmus/mon/gsn Vpu-EGFP expression plasmids in 10 cm plates, then 18-24 hrs later, seeded on coverslips as described above, and incubated at 4°C for 20 minutes. Cells were processed for surface tetherin staining by incubation with fresh media plus a polyclonal mouse anti-BST-2 antibody (Abnova, Walnut, CA) at a 1:150 dilution, at 4°C for 30 minutes, washed with PBS, fixed with 4% paraformaldehyde for 20 minutes at room temperature, and washed three times in PBS. To visualize intracellular Vpu proteins, cells were subsequently permeabilized for 10 minutes in 0.1% Triton X-100 at room temperature, washed three times in PBS and incubated with a 1:1000 dilution of rabbit anti-Vpu antiserum (ARRRP, from Frank Maldarelli and Klaus Strebel) or a 1:500 dilution of rabbit anti-GFP antibody (Invitrogen). Processed cells were mounted in Prolong Gold antifade reagent with DAPI. Images were acquired using the PerkinElmer Ultraview ERS laser spinning disk confocal imaging system at 100× magnification (PerkinElmer, Waltham, MA) and processed using Volocity software (Improvision) and Adobe Photoshop Creative Suite 2.

### Flow cytometry

HeLa cells were transfected with an EGFP expression plasmid alone (500 ng), or with 2 μg of HIV-1 Vpu or SIVmus/mon/gsn Vpu-EGFP plasmids. Twenty-four hours later, cells were blocked by incubation in 1% BSA/PBS for 20 minutes at 4°C and stained with rabbit anti-BST-2 antiserum (ARRRP, from Klaus Strebel) at a 1:5,000 dilution for 30 minutes at room temperature, followed by washing in PBS and incubation with goat anti-rabbit IgG conjugated to alexa fluor 647 (Invitrogen) at a 1:200 dilution for 20 minutes at room temperature. Cells were analyzed with a FACS CantoII (BD Biosciences, San Jose, CA) and 30,000 events were collected. Data analyses were performed using FlowJo 6.2 software (Tree Star, Ashland, OR).

## Competing interests

The authors declare that they have no competing interests.

## Authors' contributions

SJY participated in the design of the study, performed most of the experiments, and wrote the draft manuscript. LAL, HH, CME and KGH contributed to experiments and participated in the review of the manuscript. PMC conceived and coordinated the study, and wrote the final manuscript. All authors read and approved the final manuscript.
